# ALKBH3-dependent m^1^A demethylation of *Aurora A* mRNA inhibits ciliogenesis

**DOI:** 10.1038/s41421-022-00385-3

**Published:** 2022-03-11

**Authors:** Wenjun Kuang, Hao Jin, Feng Yang, Xiying Chen, Jianzhao Liu, Ting Li, Yongxia Chang, Min Liu, Zhangqi Xu, Chunxiao Huo, Xiaoyi Yan, Yuehong Yang, Wei Liu, Qiang Shu, Shanshan Xie, Tianhua Zhou

**Affiliations:** 1 The Children’s Hospital, Zhejiang University School of Medicine, National Clinical Research Center for Child Health, Hangzhou, Zhejiang China; 2 Department of Cell Biology, Zhejiang University School of Medicine, Hangzhou, Zhejiang China; 3 MOE Key Laboratory of Macromolecular Synthesis and Functionalization, Department of Polymer Science and Engineering, Zhejiang University, Hangzhou, Zhejiang China; 4 Cancer Center, Zhejiang University, Hangzhou, Zhejiang China; 5 Department of Molecular Genetics, University of Toronto, Toronto, ON Canada

**Keywords:** Cilia, RNA modification

## Abstract

Primary cilia are antenna-like subcellular structures to act as signaling platforms to regulate many cellular processes and embryonic development. m^1^A RNA modification plays key roles in RNA metabolism and gene expression; however, the physiological function of m^1^A modification remains largely unknown. Here we find that the m^1^A demethylase ALKBH3 significantly inhibits ciliogenesis in mammalian cells by its demethylation activity. Mechanistically, ALKBH3 removes m^1^A sites on mRNA of *Aurora A*, a master suppressor of ciliogenesis. Depletion of ALKBH3 enhances *Aurora A* mRNA decay and inhibits its translation. Moreover, *alkbh3* morphants exhibit ciliary defects, including curved body, pericardial edema, abnormal otoliths, and dilation in pronephric ducts in zebrafish embryos, which are significantly rescued by wild-type *alkbh3*, but not by its catalytically inactive mutant. The ciliary defects caused by ALKBH3 depletion in both vertebrate cells and embryos are also significantly reversed by ectopic expression of *Aurora A* mRNA. Together, our data indicate that ALKBH3-dependent m^1^A demethylation has a crucial role in the regulation of *Aurora A* mRNA, which is essential for ciliogenesis and cilia-associated developmental events in vertebrates.

## Introduction

m^1^A RNA modification is an important type of reversible methylation with the addition of a methyl group and a positive charge at the *N*^1^ position of adenosine^1–3^. m^1^A RNA modification is able to block the Watson-Crick interface, and thus influences RNA secondary structures and protein-RNA interactions^1,2^. m^1^A has been identified in tRNAs, rRNAs, mRNAs, and mitochondrial RNAs^2,4–9^. The dynamic methylation on RNA is mediated by effector proteins, which are termed writers (methyltransferases), erasers (demethylases) and readers (RNA binding proteins)^10^. The writers of m^1^A modification include TRMT6 (tRNA methyltransferase 6), TRMT61A, TRMT61B, and TRMT10C, while ALKBH1 (Alpha-ketoglutarate-dependent dioxygenase AlkB homolog 1) and ALKBH3 are demethylases^1^. Emerging evidence shows that m^1^A RNA modification plays a key role in RNA metabolism and gene expression^1,2,4–9^. However, the physiological function of m^1^A modification remains unclear.

Cilia are microtubule-based hair-like structures protruding from vertebrate cell surface, which transduce extracellular signals into cellular responses^11–13^. Cilia have been well demonstrated to be essential for many developmental processes^14^. Disruption of cilia structure or function causes numerous human diseases, termed ciliopathies, including polycystic kidney disease, Bardet-Biedl syndrome, and nephronophthisis^15–17^. Nevertheless, the role of m^1^A RNA modification in ciliogenesis remains unexplored.

Primary cilia occur in G0 or early G1 phase and disappear before cells enter mitosis^18^. Primary cilia assembly involves several successive stages, including maturation of the mother centriole into a basal body, formation of ciliary vesicle, recruitment of tau-tubulin kinase 2, removal of centriolar coiled-coil protein 110, assembly of transition zone, and elongation of microtubule axoneme^19–21^. Cilia disassembly is mainly regulated by the Aurora A-HDAC6 (histone deacetylase 6) pathway and the NEK2 (NIMA-related kinase 2)-KIF24 (kinesin family member 24) pathway. At early G1 phase, Aurora A is activated and recruited at basal body, and then phosphorylates and activates HDAC6, which deacetylates and destabilizes axonemal tubulins, to facilitate ciliary disassembly^22^. In S/G2 phases, NEK2 phosphorylates and activates the microtubule depolymerizing kinesin KIF24 to block re-ciliation^23^.

Here, we find that the m^1^A demethylase ALKBH3 functions as a negative regulator of ciliogenesis by removing m^1^A sites on *Aurora A* mRNA, a key regulator of cilia disassembly. Moreover, we also identify that Alkbh3 is essential for cilia-associated developmental processes in zebrafish by regulating *Aurora A* mRNA stability. Collectively, these data suggest that the ALKBH3-Aurora A axis plays a crucial role in regulating ciliogenesis and cilia-related embryonic events in vertebrates.

## Results

### ALKBH3 inhibits ciliogenesis in an m^1^A-dependent manner

**Fig. 1:**
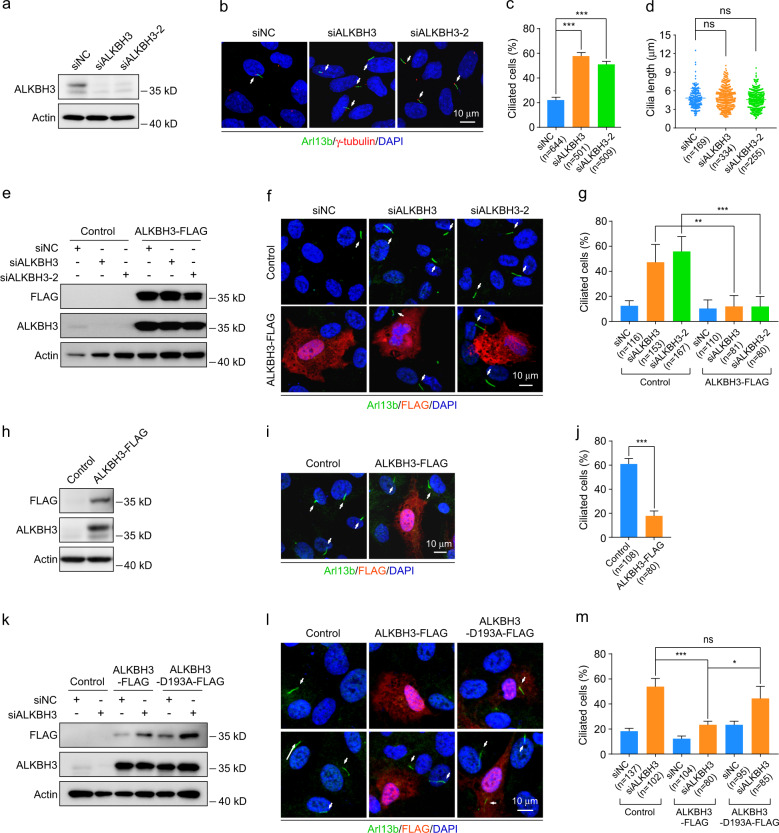
ALKBH3 inhibits ciliogenesis through its m^1^A demethylation activity. **a**–**d** RPE-1 cells were transfected with control or ALKBH3 siRNAs for 48 h in DMEM/F12 medium with 10% serum (normal conditions) and then subjected to western analysis or immunofluorescence. Western blotting of ALKBH3 protein (**a**). Representative images of RPE-1 cells with anti-Arl13b (green) and γ-tubulin (red) antibodies (**b**). Quantification analysis of the percentage of ciliated cells (**c**). Cilia length was determined by Image J software (**d**). **e**–**g**, **k**–**m** RPE-1 cells treated with the indicated siRNAs for 24 h were transfected with control, ALKBH3-FLAG, or ALKBH3-D193A-FLAG plasmid for another 24 h in normal conditions, and then subjected to western analysis or immunofluorescence. **h**–**j** RPE-1 cells transfected with the indicated plasmids for 24 h in normal conditions were treated with serum starvation for another 24 h and then applied for western analysis or immunofluorescence. Western blotting of FLAG and ALKBH3 protein was presented (**e**, **h**, **k**). Representative confocal images of RPE-1 cells with anti-Arl13b (green) and anti-FLAG (red) antibodies were shown (**f**, **i**, **l**). The percentage of ciliated cells in control or FLAG-positive group was analyzed (**g**, **j**, **m**). Actin was served as a loading control. DNA was stained by DAPI (blue). Cilia are indicated by white arrows. Scale bars, 10 μm. n, the number of total cells calculated. Data are presented as the means ± SD from at least three independent experiments. Student’s *t-*test; ns not significant; *P < 0.05, **P < 0.01, ***P < 0.001.

To investigate the role of m^1^A RNA modification in ciliogenesis, we first performed a functional screening targeting genes encoding m^1^A-regulating proteins by using small interfering RNAs (siRNAs) in human RPE-1 (retinal pigmented epithelial 1) cells in normal conditions (DMEM/F12 medium with 10% serum). Depletion of TRMT6, TRMT61A, or TRMT61B had no significant effect on ciliogenesis in RPE-1 cells (Supplementary Fig. S1). Silencing of ALKBH1 or TRMT10C with one of the two siRNAs showed an effect on cilia ratio. Importantly, knockdown of ALKBH3 with two different siRNAs significantly increased the percentage of ciliated cells, but not influenced the length of cilia (Fig. 1a–d). Furthermore, the enhanced ciliation in ALKBH3-depleted cells was significantly reversed by ectopic expression of ALKBH3-FLAG proteins (Fig. 1e–g). To confirm the function of ALKBH3 in ciliation, we exogenously expressed ALKBH3-FLAG and found that forced expression of ALKBH3-FLAG significantly reduced ciliogenesis in RPE-1 cells treated with serum starvation (Fig. 1h–j).

Since the demethylation activity of ALKBH3 on mRNA is inhibited by the mutation of D193A^7,24^, we generated a catalytically inactive mutant of ALKBH3 (ALKBH3-D193A) to examine whether the regulation of ALKBH3 in ciliogenesis is depend on its demethylation activity. The LC-MS/MS quantification of m^1^A mRNA modification in RPE-1 cells confirmed that the ALKBH3-D193A construct we made was indeed catalytically inactive (Supplementary Fig. S2). The ciliary phenotype in ALKBH3-depleted cells was significantly restored by ectopic expression of wild-type ALKBH3-FLAG, but not by ALKBH3-D193A-FLAG mutant in RPE-1 cells (Fig. 1k–m). In addition, overexpression of ALKBH3-D193A mutant had no significant effect on ciliation in RPE-1 cells with serum deprivation (Supplementary Fig. S3). Together, these data suggest that the m^1^A RNA demethylase ALKBH3 acts as a negative regulator of ciliogenesis in mammalian cells.

### ALKBH3 knockdown leads to increased m^1^A level on *Aurora A* mRNA

**Fig. 2:**
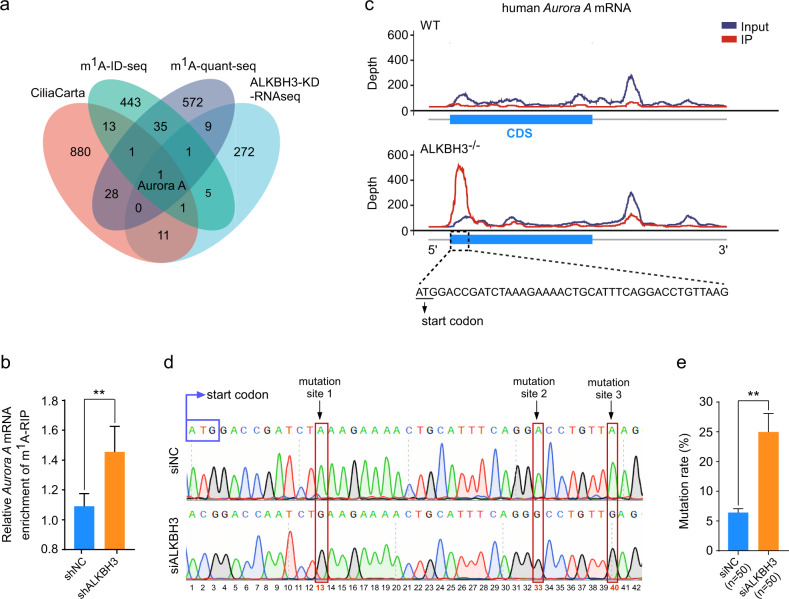
ALKBH3 removes m^1^A sites on *Aurora A* mRNA. **a** Venn diagram showing the overlapped transcripts from the databases of CiliaCarta, m^1^A-ID-seq, m^1^A-quant-seq, and RNA-seq in ALKBH3-KD cells. KD, knockdown. **b** Cells infected with lentivirus carrying shRNA targeting negative control (NC) or ALKBH3 were subjected to m^1^A RNA immunoprecipitation-qPCR analysis (m^1^A-RIP). m^1^A levels of *Aurora A* mRNA in control and ALKBH3-depleted cells were measured. **c** Peak-calling analysis of specific m^1^A peaks of *Aurora A* mRNA in wild-type and *ALKBH3* knockout HEK-293T cells. **d** Sanger sequencing of *Aurora A* PCR products amplified from cDNAs that were reverse-transcripted by RT-1306 in the control and ALKBH3-depleted cells. Mutations at the m^1^A site are highlighted with red box. **e** Mutation rates at m^1^A sites of *Aurora A* mRNA in the indicated group are shown. Data are presented as the means ± SD from at least three independent experiments. Student’s *t*-test; **P < 0.01.

To explore the molecular mechanism of ALKBH3 in ciliogenesis, we tried to identify possible mRNAs that are targeted by ALKBH3. First, we systematically analyzed the published datasets from m^1^A-ID-seq^2^ and m^1^A-quant-seq^25^ in HEK-293T cells and acquired 38 potential m^1^A-modified transcripts. These potential m^1^A-modified transcripts were further overlapped with cilia-associated genes from CiliaCarta^26^ and differentially expressed genes of RNA-seq in ALKBH3-depleted RPE-1 cells. As a result, only *Aurora A*, a master negative regulator of ciliation, was overlapped among these four datasets (Fig. 2a). To investigate if there exist m^1^A sites on *Aurora A* mRNA, a gene-specific methylated RNA immunoprecipitation-qPCR assay was performed. The data showed that the m^1^A abundance of *Aurora A* mRNA in ALKBH3-depleted cells was significantly increased when compared to that of control cells (Fig. 2b), indicating that *Aurora A* mRNAs may contain m^1^A modification.

To identify the precise m^1^A sites on *Aurora A* mRNA, we carefully analyzed the m^1^A-ID-seq dataset from wild-type and *ALKBH3* knockout HEK-293T cells^2^, and discovered there were potential m^1^A peaks located near the translation initiation region of *Aurora A* mRNA (Fig. 2c). Given that RT-1306 is an evolved HIV reverse transcriptase that able to induce mutations at m^1^A sites on RNAs^25^, we employed RT-1306 to identify the m^1^A sites on *Aurora A* mRNA. Our sequencing data from the cDNAs of control cells and ALKBH3-depleted cells revealed that there existed three m^1^A sites (+13, +33, +40) in the coding sequence region near the translation initiation site of *Aurora A* mRNA (Fig. 2d). Moreover, we also performed TA cloning from the above cDNAs amplified by RT-1306 and picked single colony for sequencing analysis. The results displayed that the mutation rate of these m^1^A sites was significantly increased in ALKBH3-depleted cells (Fig. 2e). Taken together, these observations suggest that ALKBH3 catalyzes the demethylation of m^1^A sites on *Aurora A* mRNA.

### ALKBH3 facilitates the stability and translation of *Aurora A* mRNA

**Fig. 3:**
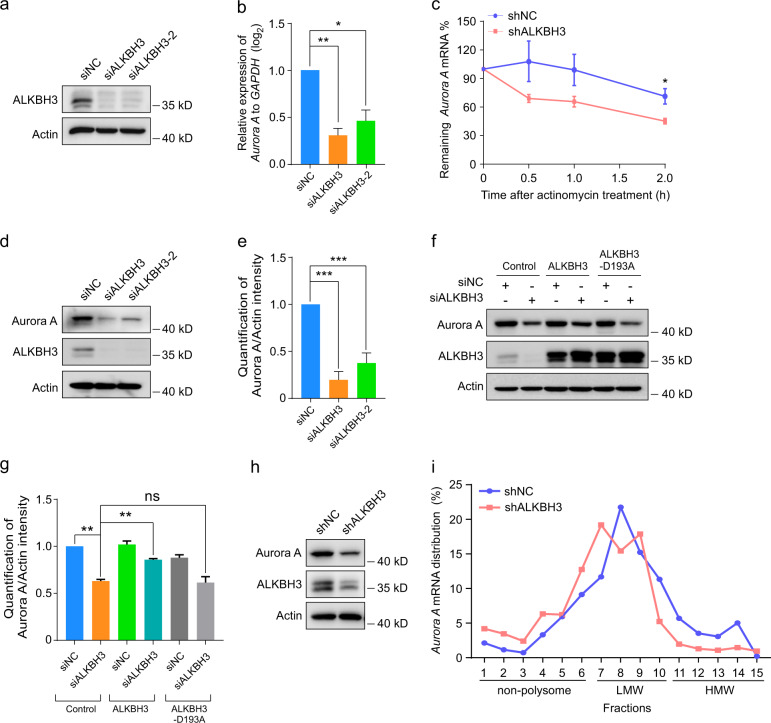
Knockdown of ALKBH3 attenuates the stability and translation of *Aurora A* mRNA. **a** Western blotting analysis of ALKBH3 in RPE-1 cells treated with the indicated siRNAs. Actin was used as a loading control. **b** Quantitative real-time PCR (qRT-PCR) analysis of *Aurora A* in RPE-1 cells transfected with the indicated siRNAs. *GAPDH* was served as an internal control. **c** Quantitative RT-PCR of *Aurora A* mRNA in ALKBH3-depleted and control cells treated with actinomycin D (5 μg/mL) at the indicated times. **d**–**g** Western blotting of Aurora A in RPE-1 cells transfected with the siRNAs or plasmids as shown. Actin, a loading control. The intensity of Aurora A was measured based on the normalization to Actin. **h** Western blotting analysis of ALKBH3 and Aurora A in RPE-1 cells infected with lentivirus carrying shRNA targeting ALKBH3. Actin was used as a loading control. NC, negative control. **i** Polysome profiling of *Aurora A* mRNA in cells infected with lentivirus carrying shRNA targeting ALKBH3 after sucrose gradient centrifugation. Data are shown as the means ± SD from at least three independent experiments. Student’s *t-*test; ns, not significant; *P < 0.05, **P < 0.01, ***P < 0.001.

Since the m^1^A modification of mRNA has been reported to influence mRNA metabolism processes^27^, including mRNA stability and translation, we first examined whether ALKBH3 regulates the mRNA levels of *Aurora A*. The results revealed that knockdown of ALKBH3 significantly decreased *Aurora A* mRNA levels in RPE-1 cells (Fig. 3a, b). Further experiments showed that the decay rate of *Aurora A* mRNA was also significantly enhanced in cells depleted of ALKBH3 when the transcription was halted with actinomycin D in cells (Fig. 3c). Next, we determined if the RNA translation efficiency of *Aurora A* mRNA is affected by ALKBH3. Western analysis showed that ALKBH3 depletion significantly decreased Aurora A protein levels, which was effectively reversed by ectopic expression of ALKBH3 but not ALKBH3-D193A mutant (Fig. 3d–g). Moreover, polysome profiling in RPE-1 cells displayed that stably knockdown of ALKBH3 resulted in reduced *Aurora A* mRNA abundance in high-molecular-weight (HMW) polysome portions, which generally have high translation efficiency^28^ (Fig. 3h, i), implying that depletion of ALKBH3 may suppress the translation of *Aurora A* mRNA. Collectively, these data suggest that ALKBH3 is essential for the regulation of the stability and translation of *Aurora A* mRNA.

### ALKBH3 inhibits ciliogenesis through Aurora A

**Fig. 4:**
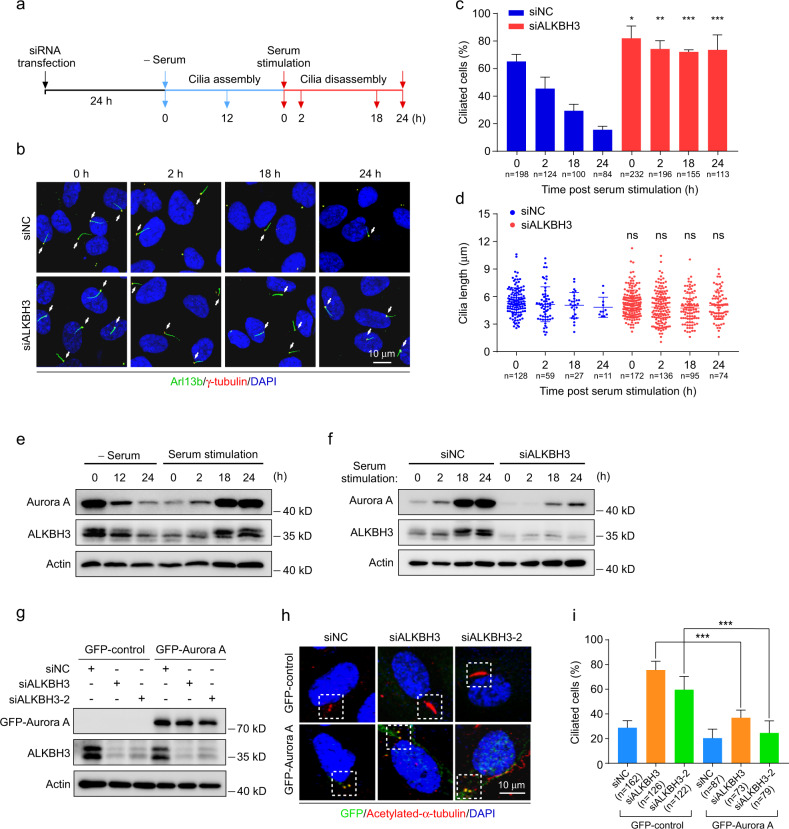
Ectopic expression of Aurora A reverses the ciliary defects in ALKBH3-depleted cells. **a**–**f** RPE-1 cells transfected with the indicated siRNAs or not were treated with serum starvation for 24 h and serum stimulation for another 24 h, and then subjected to the following analyses at the indicated time points. Schematic illustration of experimental strategy used for the cilia assembly and disassembly experiments (**a**). Representative confocal images of RPE-1 cells with anti-Arl13b (green) and γ-tubulin (red) antibodies (**b**). Cilia are indicated by white arrows. Quantification analysis of the percentage of ciliated cells (**c**). Cilia length was determined by Image J software (**d**). Western blotting analysis of Aurora A and ALKBH3 proteins at the indicated time points (**e**, **f**). Actin was served as a loading control. **g**–**i** RPE-1 cells treated with the indicated siRNAs for 24 h, followed by infection with lentivirus carrying GFP-control or GFP-Aurora A for another 48 h in normal conditions, and then subjected to western analysis or immunofluorescence. Western blotting of GFP-Aurora A and ALKBH3 protein. Actin, a loading control (**g**). Representative confocal images of RPE-1 cells with anti-GFP (green) and anti-acetylated-α-tubulin (red) antibodies (**h**). The percentage of ciliated cells in GFP-control or GFP-Aurora A-positive group (**i**). DNA was stained by DAPI (blue). Scale bars, 10 μm. n, the number of total cells calculated. Data are shown as the means ± SD from at least three independent experiments. Student’s *t-*test; ns, not significant; *P < 0.05, **P < 0.01, ***P < 0.001.

Given that Aurora A is a key regulator to facilitate cilia disassembly^19,21,29–31^, we reasoned that ALKBH3 may affect cilia disassembly. To verify this, RPE-1 cells were treated with serum starvation for 24 h to induce ciliation, and then stimulated with serum for another 24 h to trigger cilia disassembly (Fig. 4a). Under this condition, we observed a dramatic decrease of cilia in control RPE-1 cells after serum stimulation for 24 h (Fig. 4b–d). However, ALKBH3-depleted cells were significantly resistant to serum-induced cilia disassembly. Thus, these data suggest that ALKBH3 functions to regulate cilia disassembly.

Since the protein levels of Aurora A are highly dynamic during ciliary cycle^29^, we then observed the profiles of ALKBH3 and Aurora A during serum starvation/re-stimulation. Our data showed that total levels of Aurora A were slightly increased at 2 h, and peaked at 18 h and 24 h after serum stimulation (Fig. 4e), which was consistent with the previous report^29^. We also found the similar expression pattern of ALKBH3 as Aurora A during serum starvation/re-stimulation (Fig. 4e). Of note, Aurora A protein levels in ALKBH3-depleted RPE-1 cells were obviously reduced throughout serum stimulation (Fig. 4f), implying that the regulation of Aurora A expression by ALKBH3 may happen throughout ciliary cycle.

Given that the phosphorylation of Aurora A at the centrosomes is sufficiently to induce disassembly of cilia^21^, we performed immunofluorescence experiments to examine the intensity change of centrosomal Aurora A or phosphorylated Aurora A in ALKBH3 knockdown RPE-1 cells (Supplementary Fig. S4). Our data revealed that both Aurora A and phosphorylated Aurora A levels were evidently reduced in ALKBH3-depleted cells (Supplementary Fig. S4a, e). Further results showed that the fluorescence intensities of Aurora A and phosphorylated Aurora A at centrosomes were significantly decreased in cells depleted of ALKBH3 (Supplementary Fig. S4c, g). The ratio of cells with centrosomal Aurora A or phosphorylated Aurora A was also significantly reduced (Supplementary Fig. S4d, h). These observations suggest that ALKBH3 depletion attenuates the levels of centrosomal Aurora A and phosphorylated Aurora A.

In addition, silencing of Aurora A significantly promoted ciliogenesis in RPE-1 cells (Supplementary Fig. S5), which was consistent with previous report^31^. Moreover, rescue experiments showed that exogenous expression of GFP-Aurora A protein was able to significantly reverse the enhanced ciliation induced by ALKBH3 knockdown (Fig. 4g–i). These data together indicate that ALKBH3 inhibits ciliation via promoting Aurora A expression.

### ALKBH3 deficiency promotes ciliation to induce cell cycle arrest

To explore whether the enhanced ciliation in ALKBH3-depleted cells is associated with cell cycle arrest, we first examined the effect of ALKBH3 deficiency on cell cycle progression in RPE-1 cells without serum starvation. FACS analysis revealed that ALKBH3 depletion increased the percentage of cells at G0/G1 phase (Supplementary Fig. S6a, b). Immunoblotting analyses displayed that cell cycle marker cyclin A was reduced in ALKBH3-depleted cells, indicating a decrease of S or G2/M phase cells in response to ALKBH3 knockdown (Supplementary Fig. S6c)^31^. Further EdU-labeling experiments showed a significant decrease in the percentage of EdU-incorporated cells when ALKBH3 was depleted (Supplementary Fig. S6d, e). These data suggest that ALKBH3 depletion causes cell cycle arrest at G0/G1 phase.

Then, we asked whether the cell cycle arrest in G0/G1 phase induced by ALKBH3 depletion is mediated by its enhanced ciliation. Since IFT20 is a key intraflagellar transport protein required for cilia formation and has no significant effect on cell cycle progression^29,32^, we depleted IFT20 to abolish cilia growth in ALKBH3 knockdown cells to observe cell cycle progression. The data from FACS profiles, cell cycle marker, and EdU incorporation showed that cilia abrogation induced by IFT20 depletion significantly reversed the G0/G1 arrest in ALKBH3-depleted cells (Supplementary Fig. S6), indicating that ALKBH3 depletion induces G0/G1 arrest in a cilia-dependent manner.

Next, we examined whether Aurora A knockdown also led to a cilia-dependent cell cycle arrest in RPE-1 cells. Silencing of Aurora A induced cell cycle arrest at G0/G1 phase, which was also significantly reversed by simultaneous depletion of IFT20 (Supplementary Fig. S7). Collectively, these results suggest that depletion of either ALKBH3 or Aurora A causes enhanced ciliation in RPE-1, which may subsequently induce cell cycle arrest at G0/G1 phase.

### Molecular characterization of zebrafish *alkbh3*

**Fig. 5:**
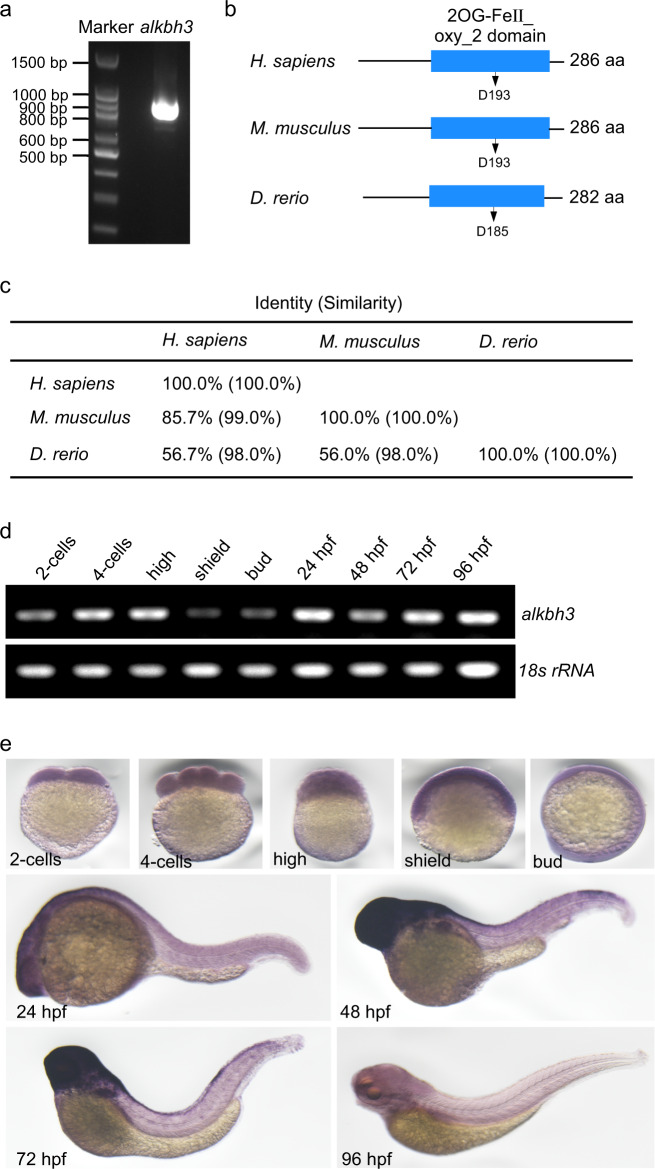
Molecular characterization of zebrafish *alkbh3* gene. **a** Cloning of zebrafish *alkbh3* by reverse transcription PCR. **b**, **c** Schematic comparison of ALKBH3 amino acid sequences from the indicated species. The conserved 2OG-Fe (II) oxygenase superfamily domains are shown in blue filled bars. The conserved amino acid residues D193 and D185 are indicated, respectively. **d** Reverse transcription PCR analysis of *alkbh3* mRNA at the different embryonic stages in zebrafish. *18s rRNA* was served as a loading control. **e** Whole-mount in situ hybridization of zebrafish *alkbh3* mRNA at the indicated developmental stages in lateral view.

To explore the role of ALKBH3 in ciliogenesis during vertebrate development, we first cloned zebrafish *alkbh3* (GenBank, NM_001003511.2) (Fig. 5a). Bioinformatics analysis revealed that *alkbh3* encoded a deduced 282 aa protein with a conserved 2OG-Fe (II) oxygenase superfamily domain^33^, implying that zebrafish Alkbh3 may also have demethylation activity (Fig. 5b). The amino acid residue D193 is critical for the demethylation activity of ALKBH3 on mRNA in *homo sapiens*^7,24^, while the residue D185 of Alkbh3 in *Danio rerio* was predicted to be evolutionarily conserved with D193. Alignment analysis showed that zebrafish Alkbh3 shared high homology with human ALKBH3 (identity, 56.7%; similarity, 98%) (Fig. 5c).

Then, we examined the temporal expression of zebrafish *alkbh3* during embryo development, and discovered that *alkbh3* mRNA was maternally deposited and ubiquitously expressed throughout early zebrafish embryonic stages (Fig. 5d). Whole-mount in situ hybridization revealed that *alkbh3* mRNA was maternally provided, enriched in brain at 48 hpf (hours post fertilization) to 72 hpf, and decreased to low levels at 96 hpf (Fig. 5e).

### *alkbh3* morphants display ciliary phenotypes in zebrafish embryos

**Fig. 6:**
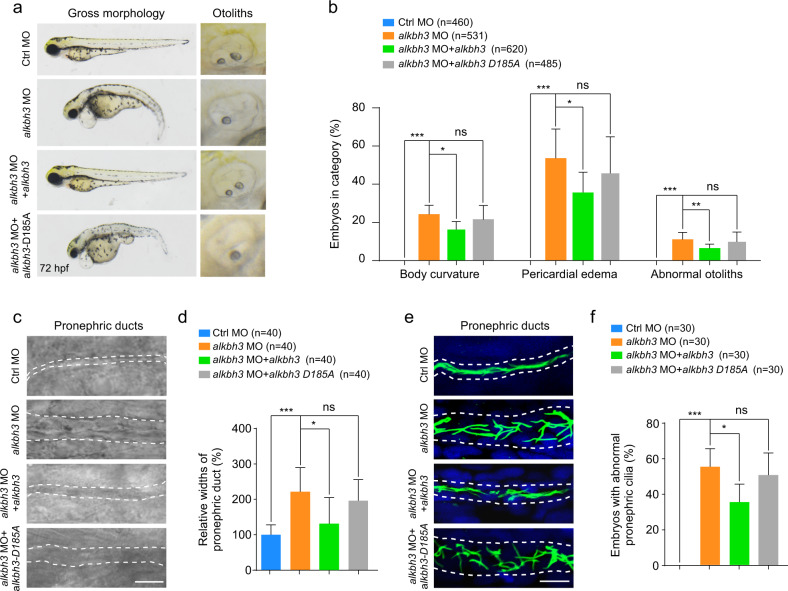
Zebrafish *alkbh3* morphants exhibit ciliary phenotypes. Zebrafish embryos were injected with control morpholino (ctrl MO) or *alkbh3* translation-block morpholino (*alkbh3* MO) at 1-cell stage and subjected to the following analyses. **a**, **b** Bright-field micrographs and statistical analysis of the ciliary phenotypes in *alkbh3* morphants. **c**, **d** Representative images, and quantification of pronephric ducts in the indicated groups. The lumen of pronephric ducts is indicated by white dashed lines. Scale bar, 10 μm. The widths of pronephric ducts in the indicated groups are measured and the median width of control morphants’ ducts was set at 100%. **e** Immunostaining of cilia in pronephric ducts in morphants. Cilia were stained by anti-acetylated-α-tubulin antibody (green). The lumen of pronephric ducts is indicated by white dashed lines. Scale bar, 10 μm. **f** Quantification of embryos with abnormal pronephric cilia. n, the total number of embryos in all experiments. Data are shown as the means ± SD from at least three independent experiments. Student’s *t-*test; ns, not significant; *P < 0.05, **P < 0.01, ***P < 0.001.

To investigate whether Alkbh3 influences ciliogenesis and embryonic development in zebrafish, we designed antisense morpholino oligonucleotides (*alkbh3* MO) to block the translation of *alkbh3* mRNA. The efficiency of *alkbh3* MO to inhibit the expression of Alkbh3-EGFP fusion protein was validated by using a reporter construct consisting of *alkbh3* MO targeting sequence fused to EGFP (Supplementary Fig. S8). *alkbh3* morphants displayed ciliary phenotypes in zebrafish embryos, including curved body, pericardial edema, abnormal otoliths in otic vesicles and dilation in pronephric ducts (Fig. 6a–d). Importantly, these defects in *alkbh3* morphants were significantly reversed by co-injection with *alkbh3* mRNA, but not *alkbh3-D185A* mRNA that was predicted to a catalytically inactive mutant of Alkbh3 based on the evolutionary conservation analysis of amino acid sequence (Fig. 5b). Further observations showed that cilia in the pronephric ducts of *alkbh3* morphants were visibly defective compared with that of control morphants at 28 hpf, which were also significantly rescued by ectopic expression of *alkbh3* mRNA rather than *alkbh3-D185A* mRNA (Fig. 6e, f). Together, these data imply that Alkbh3 may play a crucial role in cilia-associated developmental processes possibly through its demethylation activity in zebrafish.

### Ectopic expression of Aurora A reverses the ciliary defects in *alkbh3* morphants

**Fig. 7:**
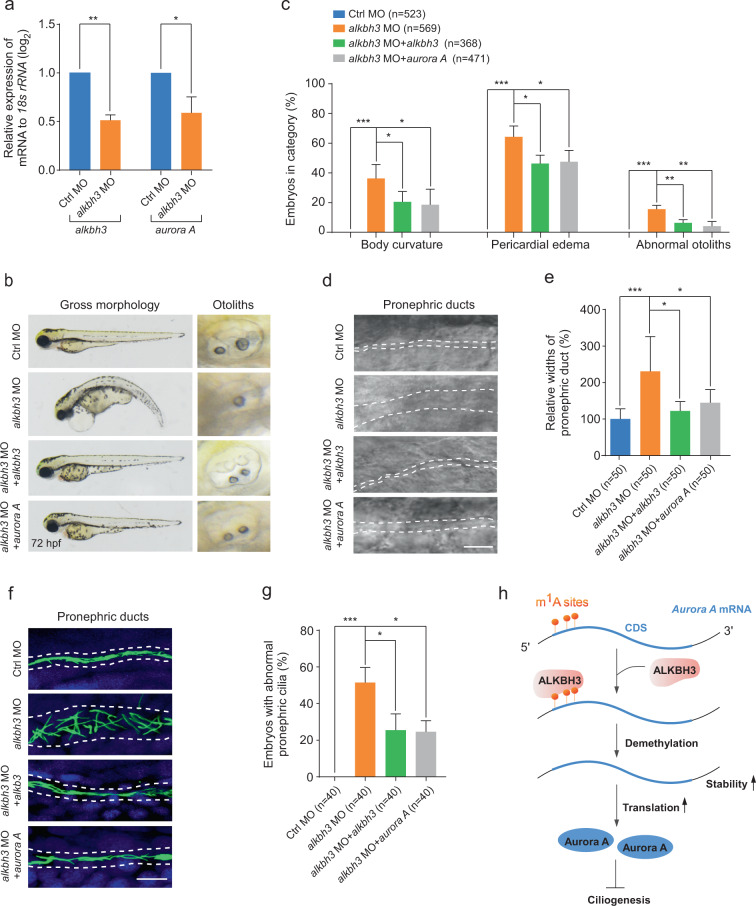
Ectopic expression of Aurora A rescues the ciliary defects in *alkbh3* morphants. Zebrafish embryos were injected with the indicated MOs or mRNAs at 1-cell stage and subjected to the following assays. **a** Quantitative RT-PCR of *aurora A* mRNA levels in *alkbh3* morphants at 48 hpf. **b**, **c** Representative images and quantification of gross morphology of embryos injected with the indicated MOs or mRNAs. **d**, **e** Brightfield images and statistical analyses of zebrafish pronephric ducts. The lumen of pronephric ducts is indicated by white dashed lines. The median width of control morphants’ ducts was set at 100%. **f** Immunofluorescence of cilia in pronephric ducts. Cilia were stained by anti-acetylated-α-tubulin antibody (green). The lumen of pronephric ducts is indicated by white dashed lines. Scale bar, 10 μm. **g** The percentage of embryos with abnormal pronephric cilia. Data are presented as the means ± SD from at least three independent experiments. Student’s *t-*test; *P < 0.05, **P < 0.01, ***P < 0.001. **h** Working model of ALKBH3-dependent m^1^A demethylation of *Aurora A* mRNA to inhibit ciliogenesis. See text for details.

To define if Aurora A is involved in the regulation of cilia-related events by Alkbh3 during zebrafish embryogenesis, we examined the expression levels of *aurora A* mRNAs in *alkbh3* morphants, and found that *aurora A* mRNA was significantly decreased in *alkbh3*-depleted embryos (Fig. 7a), which was consistent with our data from mammalian cells (Fig. 3b). Moreover, co-injection of *aurora A* mRNA was able to significantly reverse the ciliary phenotypes in *alkbh3* morphants (Fig. 7b–e). Additional data showed that ectopic expression of *aurora A* mRNA significantly rescued the abnormal ciliation in pronephric ducts in *alkbh3* knockdown embryos (Fig. 7f, g). Collectively, these results suggest that Alkbh3 influences cilia-associated developmental events possibly by regulating *aurora A* mRNA in vertebrate embryogenesis.

## Discussion

In this manuscript, our study reveals a previously uncharacterized role of the m^1^A demethylase ALKBH3 in vertebrate ciliogenesis and embryonic development. ALKBH3 removes the m^1^A modification of *Aurora A* mRNA in the coding sequence region near the translation initiation site to inhibit *Aurora A* mRNA decay and promote its mRNA translation, which consequently maintains the abundance of Aurora A protein to suppress ciliogenesis (Fig. 7h).

m^1^A RNA modification occurs in diverse RNA species and impacts RNA metabolism and gene regulation;^7,8,34^ however, its physiological consequences remain unclear. In this study, we find that depletion of *alkbh3* causes several developmental defects, including curved body, pericardial edema, abnormal otoliths, and dilation of pronephric ducts in zebrafish embryos. Moreover, these phenotypes are significantly rescued by wild-type *alkbh3*, but not its catalytically inactive mutant. Thus, our data for the first time show the biological function of m^1^A RNA modification during vertebrate embryonic development.

Few studies on the roles of RNA modification in cilia structure and function have been reported until Narry Kim’s group in 2021 showed that the m^6^A demethylase FTO (fat mass and obesity-associated protein) targets *FOXJ1* (forkhead box protein J1) mRNA to regulate proper motile ciliogenesis^35^. However, the function of m^1^A RNA modification in ciliation is still unknown. Here we identify that ALKBH3 is a negative regulator of ciliogenesis in mammalian cells. Knockdown of ALKBH3 remarkably promotes ciliation in mammalian cells, which is significantly reversed by wild-type ALKBH3, but not its catalytically inactive mutant. Collectively, these observations indicate an important role of m^1^A RNA modification in ciliation, providing a new perspective for the study of cilia regulation.

ALKBH3 is a member of Alpha-ketoglutarate-dependent dioxygenase AlkB homology family, which is able to remove alkyl adducts from nucleobases by oxidative dealkylation^36^. Previous studies have shown that ALKBH3 is an alkylation damage repair enzyme to repair m^1^A and m^3^C (3-methylcytosine) alkylation damage in RNA and ssDNA^33,37^. Emerging data demonstrate that ALKBH3 can remove the methyl group of m^1^A in mRNA and tRNA^2,7,38,39^. However, little is known about the role of ALKBH3-mediated RNA demethylation in vertebrate ciliation and development. Herein, we find that ALKBH3 influences ciliogenesis and cilia-associated developmental events by targeting *aurora A* mRNA in an m^1^A-dependent manner.

Aurora A has been demonstrated as a key protein to promote cilia disassembly^24,25^. In the initiation of ciliary disassembly, Aurora A is phosphorylated and activated at the basal body, and then phosphorylates and activates HDAC6 (histone deacetylase 6), which in turn deacetylates and destabilizes axonemal tubulin to further facilitate ciliary disassembly^21^. Human enhancer of filamentation 1, trichoplein and pitchfork have been shown to interact with Aurora A at the ciliary base and increase the level of phosphorylated Aurora A during cilia disassembly^21,31,40^. However, the post-transcriptional regulation of *Aurora A* mRNA remains unclear. In this manuscript, our data show that ALKBH3 demethylates m^1^A sites in the coding sequence region of *Aurora A* mRNA to promote its stability and translation. Future studies are clearly required to explore the molecular mechanism underlying the epigenetic regulation of Aurora A kinase.

While our study shows that ALKBH3-dependent m^1^A demethylation of *Aurora A* mRNA regulates ciliogenesis and vertebrate development, two limitations of this work should be addressed. (1) The published datasets of m^1^A-seq we employed are from 293 T cells, while RPE-1 cells are used to investigate the role of ALKBH3 in ciliogenesis in our experiments. (2) We overlapped cilia-associated genes from CiliaCarta with m^1^A-seq data to detect the potential targets that mediate the function of ALKBH3 in ciliogenesis, which may preclude finding unknown genes that regulate ciliation. Therefore, future works on systematically screening the substrates mediating the regulation of ALKBH3 in ciliogenesis are clearly needed.

## Materials and methods

### Cell culture and transfection

RPE-1 cells were maintained in DMEM/F12 (Corning, USA) supplemented with 10% FBS (fetal bovine serum, ExCell Bio, China) and antibiotics at 37 °C in 5% CO_2_. The indicated plasmids were transfected into RPE-1 cells with Lipofectamine 3000 (Invitrogen, USA) according to the manufacturer’s instructions.

### RNA extraction and quantitative real-time PCR

Total RNAs were isolated from RPE-1 cells with TRIzol according to the manufacturer’s instructions (Invitrogen, USA). DNase I was added to isolated RNAs to avoid DNA contamination. Total RNAs were then reverse transcribed into cDNA with HiScript II 1^st^ strand cDNA Synthesis kit (Vazyme, China). Quantitative RT-PCR (qRT-PCR) analysis was performed with HiScript Q RT SuperMix (Vazyme, China) using a Bio-Rad CFX-Touch System. All reactions were performed in triplicate. The primers for qRT-PCR of, *Aurora A* and *GAPDH* in RPE-1 cells are listed: *Aurora A*, 5′-AAGACTTGGGTCCTTGGGTC-3′ and 5′-TGGTTGCCTGCAATTGCTTC-3′; *GAPDH*, 5′-GAAGGTCGGAGTCAACGG-3′ and 5′-TGGAAGATGGTGATGGGAT-3′. The primers for qRT-PCR of *alkbh3*, *aurora A* and *18* *s rRNA* in zebrafish embryos are listed: *alkbh3*, 5′-GCAGCCGCTTCCTGAAGATA-3′ and 5′-TGGTGCGGAAAGTGAGGTTT-3′; *aurora A*, 5′-GTCCAACGTCCTGTTGGGAA-3′ and 5′-TGGATCACAGCCTTCGAGTG-3′; *18* *s rRNA*, 5′-CGAACGTCTGCCCTATCAACTT-3′ and 5′-ACCCGTGGTCACCATGGTA-3′.

### Plasmid constructions

Full-length human ALKBH3 and ALKBH3-FLAG was amplified from cDNAs and cloned into pCS2+ vector. The mutation of ALKBH3-D193A and ALKBH3-D193A-FLAG was produced by PCR and cloned into a pCS2+ vector. Full-length human Aurora A was amplified from cDNAs and cloned into pLVX-GFP vector to generate GFP-Aurora A. Full-length zebrafish *alkbh3*-flag or *alkbh3*-*D185A*-flag was inserted into pCS2+ to generate morpholino-resistant *alkbh3*-flag or *alkbh3*-*D185A*-flag plasmids. Full-length zebrafish *aurora A*-flag was also inserted into pCS2+ to generate pCS2-*aurora A*-flag plasmid. The reporter *alkbh3-*5′ATG-EGFP was constructed by inserting EGFP downstream of *alkbh3*-5′ATG, which harbors the morpholino-targeting sequence into pCS2+ vector under the control of CMV promoter.

### TA cloning assay

Aurora A PCR products amplified from cDNAs that were reverse-transcripted by RT-1306 in the control and ALKBH3-depleted cells were cloned into the pEASY-Blunt Zero vector according to the manufacturer’s instructions (TransGen Biotech, China).

### ShRNAs and siRNAs

For stable knockdown, shRNA oligos targeting ALKBH3 were designed and cloned into the pLKO.1 vector (Addgene, USA). The sequences of shRNA targeting ALKBH3 were listed: Forward: CCGGCGCACATTTGAGATGAGAAAGCTCGAGCTTTCTCATCTCAAATGTGCGTTTTTTG, Reverse: AATTCAAAAAACGCACATTTGAGATGAGAAAGCTCGAGCTTTCTCATCTCA AATGTGCG. Lentiviruses were packaged with pLKO.1, pCMV-8.91 and VSV-G plasmids in HEK-239T cells, and the culture medium were harvested at 48 h. The RPE-1 cells were infected with the lentiviures-containing medium for 48 h, and followed by puromycin selection for another 48 h. For transient knockdown, siRNA oligos targeting *ALKBH3* or *Aurora A* were synthesized by GenePharma, and their sequences are also listed: 5′-GAAAGAAGCUGACUGGAUATT-3′ (si*ALKBH3*), 5′-GAGAGAAGCUUCACUGAAATT-3′ (si*ALKBH3*-2), 5′-GAAAGCUCCACAUCAAUAATT-3′ (si*Aurora A*), and 5′-CAGCAACUUCAAGCCCUAAUA-3′ (si*IFT20*). The indicated siRNAs were transfected with Lipofectamine iMAX (Invitrogen, USA) according to the manufacturer’s instructions.

### Antibodies

The following antibodies were commercially acquired for western blotting analysis: ALKBH1 (rabbit, 1:1000, Proteintech, 27973-1-AP), ALKBH3 (rabbit, 1:1000, Abcam, ab227496), Aurora A (rabbit, 1:1000, Cell Signaling, 14475), phospho-Aurora A (Thr288) (rabbit, 1:1000, Cell Signaling, 3079), Cyclin A (rabbit, 1:1000, Proteintech, 18202-1-AP), FLAG (mouse, 1:1000, Sigma, F1804), GFP (mouse, 1:1000, ABclonal, AE012), TRMT6 (rabbit, 1:1000, Proteintech, 16727-1-AP), TRMT61A (rabbit, 1:1000, Absin, abs133999), TRMT61B (rabbit, 1:1000, proteintech, 26009-1-AP), TRMT10C (rabbit, 1 μg/mL, Genetex, GTX47292), β-actin (mouse, 1:2000, Proteintech, 66009-1-Ig) and goat anti-mouse (1:5000, ABclonal, AS003) or anti-rabbit-IgG-HRP (1:5000, ABclonal, AS014).

The following antibodies were used for immunofluorescence analyses: acetylated α-tubulin (mouse, 1:1000, Sigma, T7451), Arl13b (rabbit, 1:200, Proteintech, 17711-1-AP), γ-tubulin (mouse, 1:1000, Sigma, T6557), Aurora A (rabbit, 1:100, Cell Signaling, 14475), phospho-Aurora A (Thr288) (rabbit, 1:50, Cell Signaling, 3079), FLAG (mouse, 1:200, Sigma, F1804), GFP (rabbit, 1:200, Genscript, A01388) and Alexa Fluor 488-, 568-, and 647-conjugated anti-rabbit or anti-mouse IgGs (1:200, Invitrogen).

Anti-m^1^A antibody (mouse, 1 μg/mL, MBL, D345-3) and mouse IgG (1 μg/mL, Santa Cruz biotechnology, sc-2025) were commercially acquired for m^1^A RNA immunoprecipitation.

### Western blot analysis

Western blot assays were performed as described previously^41^. Cells were lyzed on ice in TBSN buffer (20 mM Tris, pH 8.0, 150 mM NaCl, 0.5% NP-40, 5 mM EGTA, 1.5 mM EDTA, 0.5 mM Na_3_VO_4_, 20 mM p-nitrophenyl phosphate) with protease inhibitor cocktails (Roche, Switzerland) for 30 min. The protein samples were separated by SDS-PAGE, and then transferred to polyvinylidene fluoride membranes (Millipore, USA). The membranes were blocked with 5% non-fat milk for 1 h at room temperature and then incubated with the corresponding primary antibodies overnight at 4 °C. After washing, the membranes were incubated with HRP-conjugated secondary antibodies for 1 h at room temperature. Protein bands were detected using ChemiDoc Touch Imaging System (Bio-Rad, USA).

### Immunofluorescence analysis

Immunofluorescence assays were carried out as described previously^42,43^. Cells grown on coverslips were fixed with 100% methanol for 10 min at –20 °C and blocked with 5% BSA in 0.1% PBST (0.1% TritonX-100 in phosphate-buffered saline buffer) for 1 h. Cells were then incubated with the indicated primary antibodies overnight at 4 °C, followed by species-specific Alexa Fluor 488-, 555-, and 647- conjugated secondary antibodies (Invitrogen, USA) for 1 h. Nuclei were stained with DAPI (Sigma-Aldrich, USA). Images were acquired by using a laser scanning confocal microscope (OLYMPUS FV3000 OSR).

### RNA-seq

RPE-1 cells were transfected with control or ALKBH3 siRNA for 48 h in DMEM/F12 medium with 10% serum. Total RNAs were extracted from control and ALKBH3-depleted cells and then subjected to library construction and sequencing (Novagene company, China).

### m^1^A RNA immunoprecipitation

Total RNAs were extracted from RPE-1 cells with TRIzol reagent followed by an additional DNase I treatment to avoid DNA contamination. 40 µg total RNAs were then incubated with 1 μg anti-m^1^A antibody in RIP buffer (150 mM NaCl, 0.1% NP-40, 10 mM Tris, pH 7.4) at 4 °C overnight. After incubation, 40 μl Protein A/G beads rinsed with RIP buffer were added to the mixture of RNA and antibody and incubated for an additional 4 h at 4 °C. Beads were washed five times with IPP buffer and precipitated RNAs were further purified with TRIzol according to the manufacturer’s instructions (Invitrogen, USA). Input and immunoprecipitated RNAs were reverse transcribed into cDNAs and quantified by qRT-PCR.

### mRNA decay assay

RPE-1 cells were treated with 5 μg/mL actinomycin D (MedChemExpress, USA) to suppress global mRNA transcription. Cells were harvested at different time points and total RNAs were extracted for reverse transcription. The levels of *Aurora A* mRNA after transcription inhibition were detected by qRT-PCR.

### Polysome fractionation

Polysome fractionation was performed as described previously^28,44^. Briefly, ALKBH3-depleted or control RPE-1 cells (four 150-mm culture dishes) were treated with 10 μg/mL cycloheximide (MedChemExpress, USA) for 5 min at 37 °C. Then, cells were harvested and 500 μl of cytoplasmic extract was layered onto 11 mL of 5%–50% sucrose gradient and centrifuged at 36,000 rpm in a Beckman SW-41Ti rotor for 2 h at 4 °C. Gradients were fractionated and monitored at absorbance 254 nm (Brandel, USA). Polysome-associated and cytosolic RNAs of each fraction were isolated by using TRIzol and analyzed by qRT-PCR.

### Flow cytometry analysis

For flow cytometry analysis, RPE-1 cells were collected, washed once with cold phosphate-buffered saline, and then fixed in 70% ethanol. DNA was stained with 100 μg/mL propidium iodide and 50 μg/mL RNase A for 30 min at 37 °C. The samples were analyzed using an FC 500 MCL Flow Cytometer (Beckman Coulter).

### EdU assay

RPE-1 cells incubated with 50 μM EdU (5-Ethynyl-2′-deoxyuridine) for 2 h were fixed for 30 min in 4% PFA. EdU staining was then performed using the Cell-Light EdU Apollo567 In Vitro Kit according to the manufacturer’s instructions (RIBOBIO, China).

### Zebrafish maintenance and manipulations

Wild-type zebrafish (strain AB) was maintained at 28.5 °C using standard protocols. Zebrafish experiments were performed according to the requirements by Regulation for the Use of Experimental Animals in Zhejiang Province (ETHICS CODE Permit NO. ZJU2011-1-11-009Y). Synthetic capped mRNAs were transcribed in vitro using linearized zebrafish pCS2-*alkbh3*-flag, pCS2-*alkbh3*-*D185A*-flag, or pCS2-*aurora A*-flag plasmids with the MessageMachine Kit (Ambion, USA). *alkbh3* MO (5′-GCTGCCTTTTATCGCTCATCTAAAG-3′) was synthesized by GeneTools to block the translation of *alkbh3* mRNA. *alkbh3* MO (0.3 nmol/μL) or control MO (0.3 nmol/μL) was injected into zebrafish embryos at 1-cell stages. For rescue experiments, *alkbh3* mRNA (2 ng/μL), *alkbh3-D185A* mRNA (2 ng/μL), and *aurora A* mRNA (5 ng/μL) were co-injected with *alkbh3* MO at 1-cell stages, respectively.

### Whole-mount in situ hybridization

Whole-mount in situ hybridization was carried out as described previously^45^. Antisense RNA probes for *alkbh3* mRNA were labeled with digoxigenin (Roche, Switzerland). Zebrafish embryos at different stages were fixed in 4% paraformaldehyde and incubated with digoxigenin-labeled RNA probes. Alkaline phosphatase-coupled anti-digoxigenin antibody (Roche, Switzerland) was used to detect hybridized probes, and 5-bromo-4-chloro-3-indolyl phosphate (BCIP)/nitro blue tetrazolium (NBT) solution (Sigma, USA) was used as the chromogenic substrate.

### Whole-mount immunofluorescence microscopy

Whole-mount immunofluorescence was performed as described previously^45^. Zebrafish embryos were fixed in 4% paraformaldehyde overnight at 4 °C. Fixed embryos were washed with washing buffer (0.1% Triton X-100, 0.2% DMSO, and 5% bovine serum albumin in PBS buffer) for 15 min, and then blocked with blocking buffer (0.1% Triton X-100, 0.2% DMSO, 10% goat serum and 5% bovine serum albumin in PBS buffer) for 1 h at room temperature. Embryos were then incubated with primary antibodies diluted in blocking buffer overnight at 4 °C, followed by secondary antibodies (Invitrogen, USA) overnight at 4 °C. Nuclei were stained with DAPI (Sigma-Aldrich, USA). Samples were rinsed with washing buffer, placed in PBS in a 35 mm petri dish, and examined using 40× water-immersion objective lenses by confocal laser scanning microscopy (Olympus BX61W1-FV1000).

### Peak-calling

The raw data of m^1^A-ID-seq were obtained from NCBI Gene Expression Omnibus (accession number GSE73941). Reads were trimmed by Trim_galore (http://www.bioinformatics.babraham.ac.uk/projects/trim_galore/) and aligned to human genome (hg19) with HISAT2 program (http://daehwankimlab.github.io/hisat2/). Sam/bam files were processed by SAMtools (http://samtools.sourceforge.net/) as previously described^2^.

### Statistics

Data are representative of at least three independent experiments. Means and standard deviations (SD) were calculated and shown in the graphs. Student’s *t-*test was performed using GraphPad Prism software. A value of *P* < 0.05 was considered statistically significant.

## Supplementary information

Supplemental Figs.1-8
