# Supplementary material for: ALKBH3-dependent m1A demethylation of Aurora A mRNA inhibits ciliogenesis
Source: Cell Discov. 2022 Mar 11;8:25. doi: 10.1038/s41421-022-00385-3 (PMC8917145; doi:10.1038/s41421-022-00385-3)
Supplement: Supplementary file 1 — Supplemental Figs.1-8 [file 41421_2022_385_MOESM1_ESM.pdf]

# Supplementary Information

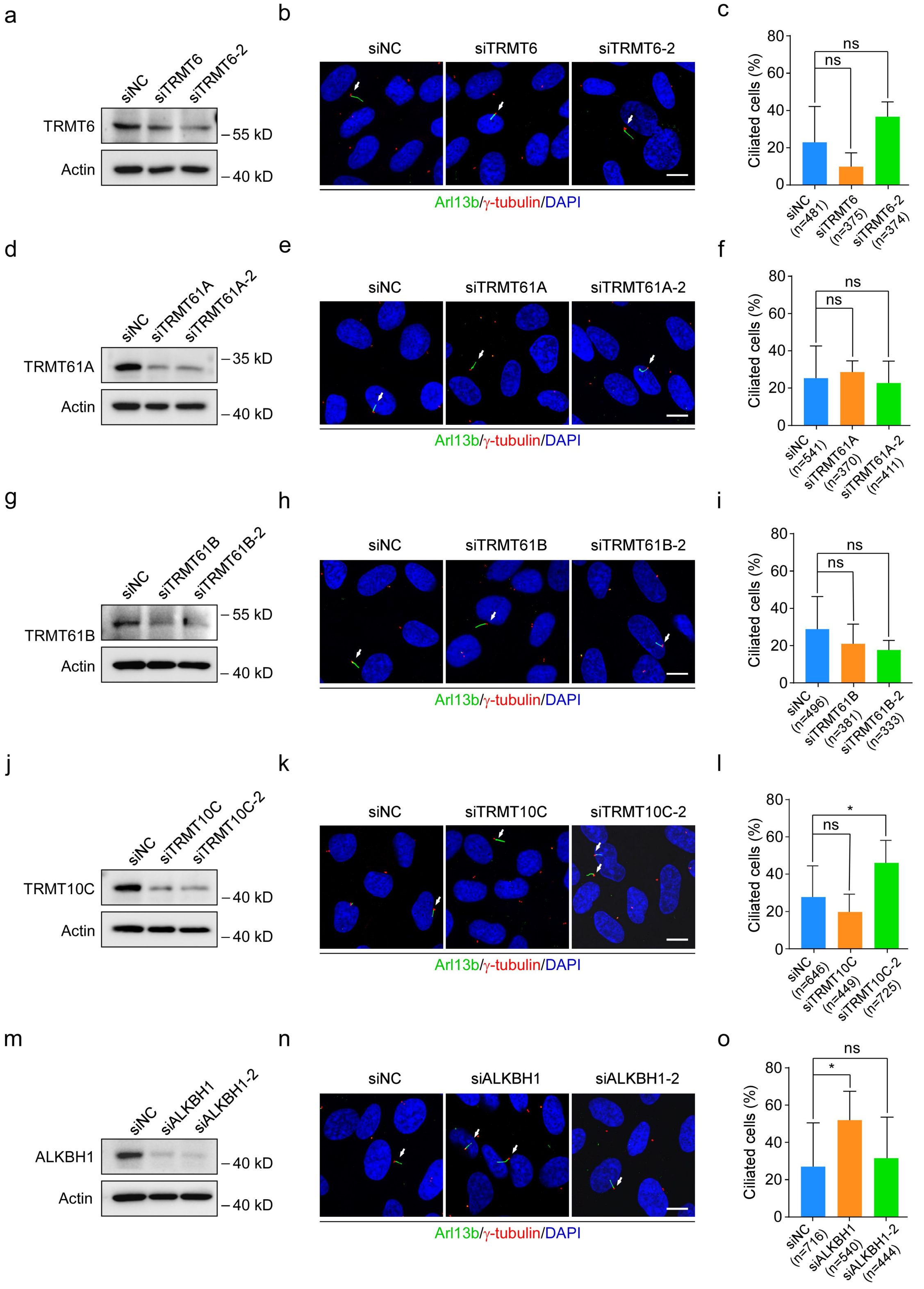


**Supplementary Fig. S1 The role of m$^1$A RNA modifying proteins in ciliogenesis.**

RPE-1 cells were transfected with the indicated siRNAs for 48 h in normal conditions

(DMEM/F12 medium with 10% serum), and then subjected to western analysis and immunofluorescence. **a, d, g, j, m** Western blotting of TRMT6 (tRNA methyltransferase 6), TRMT61A, TRMT61B and TRMT10C, and ALKBH1 (AlkB homolog 1, histone H2A dioxygenase). Actin was served as a loading control. **b, e, h, k, n** Representative confocal images of RPE-1 cells with the antibodies against Arl13b (green) and γ-tubulin (red). DNA was visualized with DAPI (blue). Cilia are indicated by white arrows. Scale bar, 10 μm. **c, f, i, l, o** Statistic analyses of the percentage of ciliated cells. n, the number of total cells calculated. Data are expressed as the means±SD from three independent experiments. Student's *t* test; ns, not significant; $*P < 0.05$.

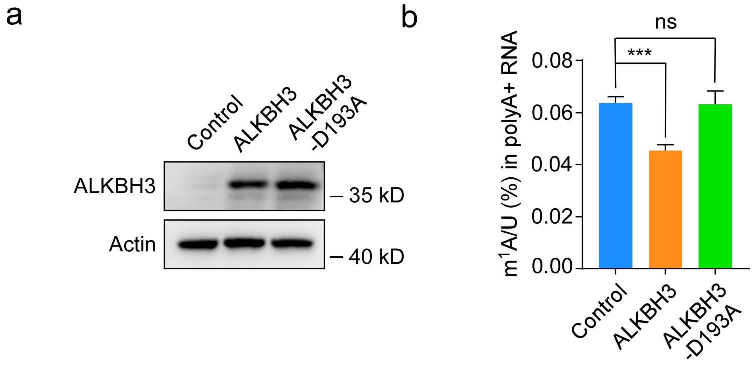


**Supplementary Fig. S2 LC-MS/MS quantitation of m$^1$A modification in mRNA.** RPE-1 cells transfected with the indicated plasmids for 48 h in normal conditions were subjected to LC-MS/MS analysis. **a** Western blotting of the ALKBH3 protein. Actin was used as a loading control. **b** LC-MS/MS quantitation of m$^1$A modification in mRNA isolated from RPE-1 cells. Data are shown as the mean±SEM (three independent experiments). Student's *t* test; ns, not significant; ***$P < 0.001$.

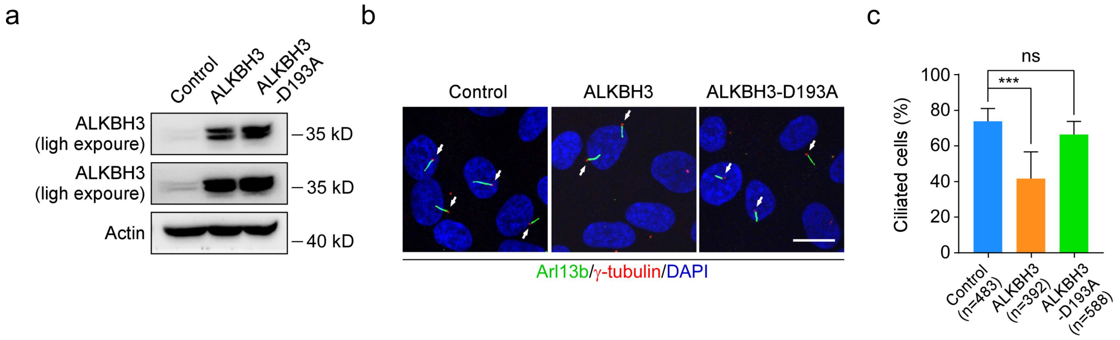


**Supplementary Fig. S3 Ectopic expression of ALKBH3 catalytic mutants has no effect on ciliation.** RPE-1 cells were transfected with the indicated plasmids for 24 h in normal conditions, and then processed for serum starvation for 24 h. **a** Western blotting of ALKBH3 protein was presented. Actin, a loading control. **b** Representative confocal images of RPE-1 cells with anti-Arl13b (green) and γ-tubulin (red) antibodies were shown. DNA was stained by DAPI (blue). Cilia are indicated by white arrows. Scale bar, 10 µm. **c** The percentage of ciliated cells was calculated. n, the number of total cells calculated. Data are presented as the means±SD from at least three independent experiments. Student's *t* test; ns, not significant; ***$P < 0.001$.

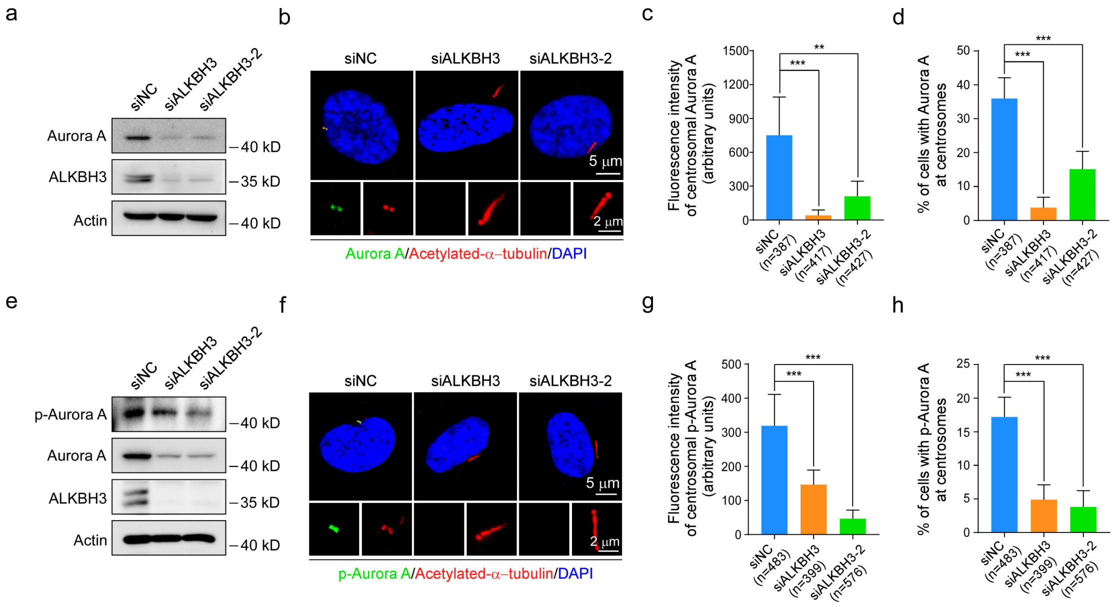


**Supplementary Fig. S4 Centrosomal Aurora A and phosphorylated Aurora A are reduced in ALKBH3-depleted cells.** RPE-1 cells were transfected with the indicated siRNAs for 48 h in normal conditions, and then subjected to western analysis or immunofluorescence. **a, e** Western blotting of ALKBH3, Aurora A and phosphorylated Aurora A (p-Aurora A) in RPE-1 cells. Actin, a loading control. **b, f** Immunofluorescence of RPE-1 cells with the indicated antibodies. Scale bar, 2 or 5 μm. **c, g** Quantification of the fluorescence intensity of Aurora A or p-Aurora A at centrosomes. Fluorescence intensity was determined by Image J software. **d, h** Quantification of the percentage of cells with Aurora A or p-Aurora A at centrosomes. n, the number of total cells calculated. Data are presented as the mean±SD from at least three independent experiments. Student's *t* test; $^{**}P < 0.01$, $^{***}P < 0.001$.

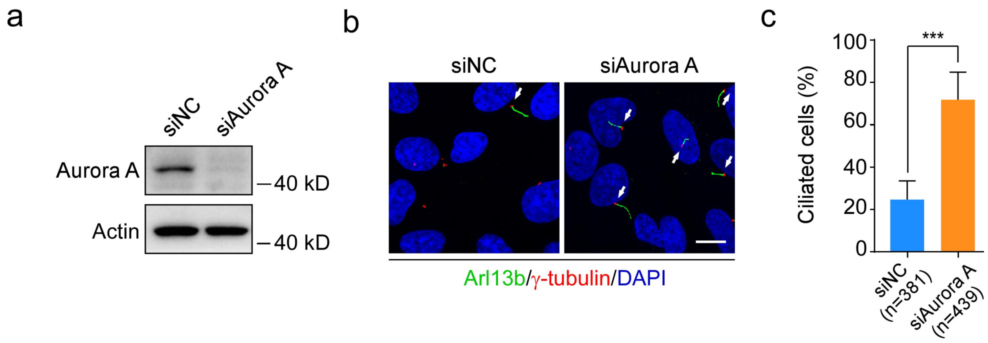


**Supplementary Fig. S5 Depletion of Aurora A suppresses ciliogenesis.** RPE-1 cells were transfected with control or Aurora A siRNA for 48 h in normal conditions, and then applied for western analysis or immunofluorescence. **a** Western blotting of Aurora A protein. Actin was served as a loading control. **b** Representative images of RPE-1 cells with anti-Arl13b (green) and γ-tubulin (red) antibodies. DNA was visualized by DAPI (blue). Scale bar, 10 µm. **c** Quantification analysis of the percentage of ciliated cells. n, the number of total cells calculated. Data are presented as the mean±SD from at least three independent experiments. Student's *t* test; ***$P < 0.001$.

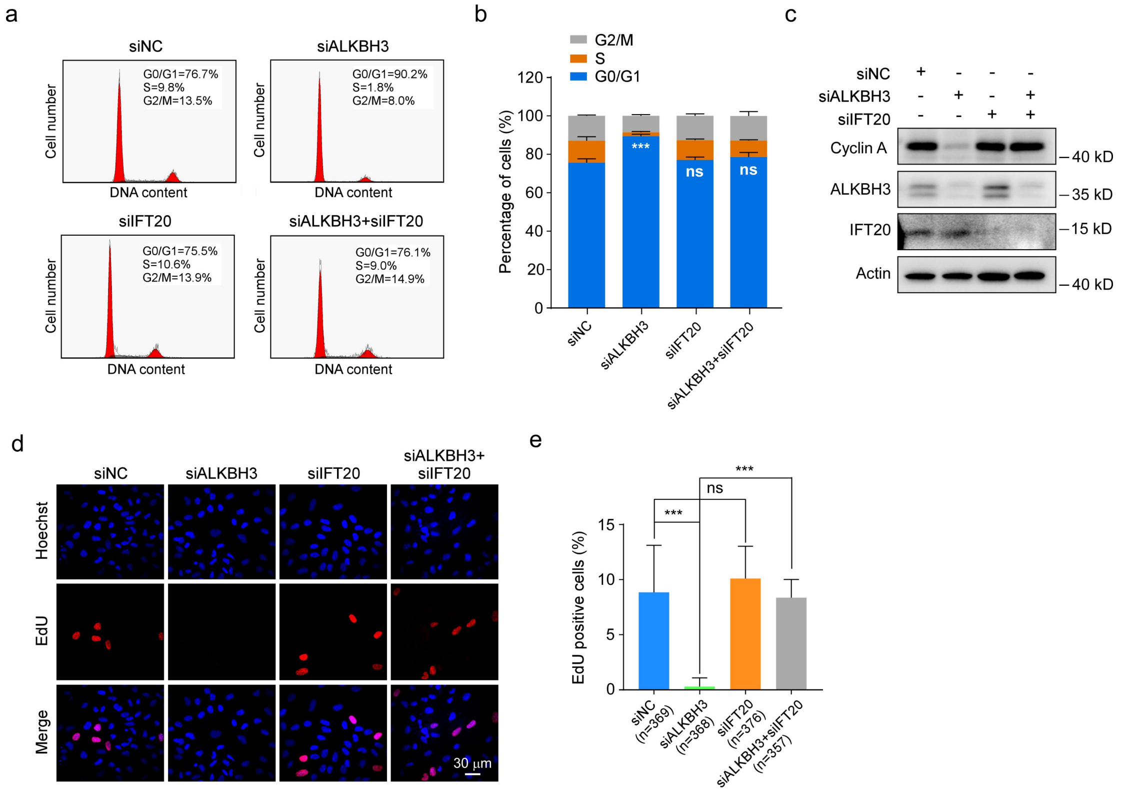


**Supplementary Fig. S6 ALKBH3 knockdown induces a cilia-dependent cell cycle arrest in G0/G1 phase.** RPE-1 cells were transfected with the indicated siRNAs for 48 h in normal conditions, and then processed to the following analyses. **a, b** FACS analysis using propidium iodide staining. Cell populations in G0/G1, S, and G2/M phases are given as percentages of total cells. Graphical representations of the cell cycle distribution are shown. **c** Immunoblotting of the indicated proteins. Actin, a loading control. **d** Cells were stained with EdU (red) and DNA (blue). Scale bar, 30 μm. **e** The percentages of EdU-positive cells were calculated. n, the number of total cells calculated. Data are presented as the mean±SD from at least three independent experiments. Student's *t* test; ns, not significant; ****P* < 0.001.

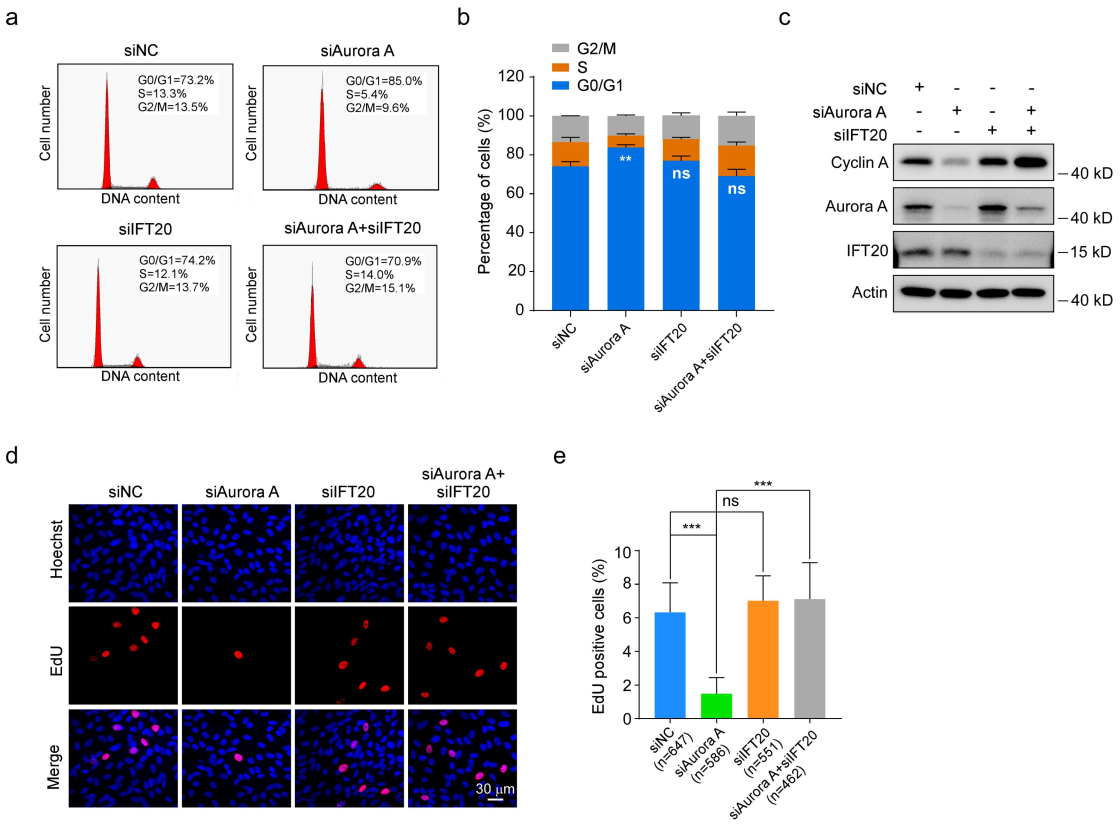


**Supplementary Fig. S7 Depletion of Aurora A results in a cilia-dependent G0/G1 phase arrest.** RPE-1 cells were transfected with the indicated siRNAs for 48 h in normal conditions, and then subjected to the following analyses. **a, b** FACS analysis using propidium iodide staining. Cell populations in G0/G1, S, and G2/M phases are given as percentages of total cells. Graphical representations of the cell cycle distribution are shown. **c** Western blots of the indicated proteins. Actin was served as a loading control. **d** Cells were stained with EdU (red) and DNA (blue). Scale bar, 30 µm. **e** The percentages of EdU-positive cells were quantified. n, the number of total cells calculated. Data are presented as the mean±SD from at least three independent experiments. Student's *t* test; ns, not significant; $**P < 0.01$, $***P < 0.001$.

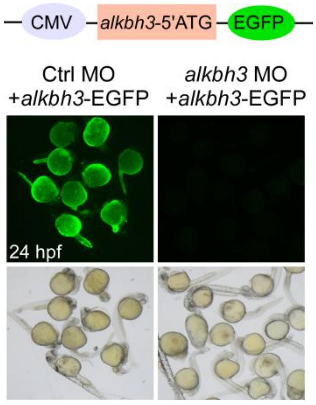


**Supplementary Fig. S8 The knockdown efficiency of *alkbh3* morpholinos.** Representative images of the embryos co-injected the indicated construct with control MO or *alkbh3* MO at 24 hpf. The diagram of the construct containing *alkbh3*-5' ATG fused with EGFP is shown.
